# Analytical study examining hypertension and arrhythmia among shift workers in healthcare

**DOI:** 10.6026/973206300212901

**Published:** 2025-08-31

**Authors:** Rahi Sutaria, Akshita Tandon, Elangovan Raman, Ajeet Saoji, Ridhay J.

**Affiliations:** 1 Department of Pharmacology, GMERS, Himmatnagar, Gujarat, India; 2 Department of Gastroenterology, Nottingham University Hospital, Nottinghamshire, UK; 3 Department of Internal Medicine, Institute of Internal Medicine, Madras Medical College, Chennai, Tamil Nadu, India; 4 Department of Community Medicine, NKP Salve Institue of Medical Sciences & Research Centre, Nagpur, Maharashtra, India; 5 Department of Cardiology, Manipal Hospital Millers Road, Karnataka, India

**Keywords:** Hypertension, arrhythmia, shift work, healthcare workers, circadian disruption, occupational health

## Abstract

The prevalence of hypertension and arrhythmia among 140 healthcare shift workers aged 25-60 years is of interest. Data on blood pressure, ECG findings, work schedules and lifestyle factors were collected and analyzed. Night shift workers showed a significantly higher prevalence of both conditions compared to day workers. Duration and frequency of night shifts were positively correlated with cardiovascular risk. Thus, we show occupational scheduling as a modifiable risk factor in healthcare-associated cardiovascular disease.

## Background:

Shift work is an integral component of healthcare systems, ensuring round-the-clock patient care, but it comes with considerable health consequences for workers [1]. Disruption of circadian rhythms due to night and rotating shifts has been linked to adverse cardiovascular outcomes, including elevated blood pressure and cardiac arrhythmias [2]. Healthcare workers often exposed to chronic stress, irregular sleep patterns, and limited recovery periods, are especially vulnerable to these risks [3]. Hypertension and arrhythmias are major predictors of cardiovascular morbidity and mortality, and their prevalence appears to be higher among populations exposed to circadian misalignment [4]. However, limited research has specifically focused on healthcare shift workers, a population uniquely affected by prolonged exposure to night shifts and high-stress environments [5]. While some studies suggest a direct link between shift work and elevated cardiovascular risk, evidence is still evolving and often confounded by factors such as lifestyle, sleep quality and job role [6]. Therefore, it is of interest to analyze and quantify the prevalence of hypertension and arrhythmias among healthcare shift workers and assess how work patterns particularly night shift frequency and duration correlate with these conditions.

## Materials and Methods:

140 healthcare professionals, ages 25 to 60, who had worked continuously for at least a year at a tertiary care hospital, participated in this analytical cross-sectional study. Based on their work schedules, participants were divided into two groups: rotating/night shift workers (n=75) and fixed day shift workers (n=65). Diagnostic tests, physical examinations and structured questionnaires were used to gather data. The Pittsburgh Sleep Quality Index (PSQI) was used to measure sleep quality, demographic information, shift schedule history and lifestyle choices (diet, exercise, smoking, and caffeine consumption). After ten minutes of rest, blood pressure was taken with a calibrated automated sphygmomanometer and hypertension was categorized using the American Heart Association's recommendations. Every participant had a 12-lead ECG, which was evaluated by a qualified cardiologist to look for arrhythmias. The primary outcomes were the prevalence of hypertension and arrhythmias across shift types and secondary outcomes included associations with shift duration (years in night shift work), frequency (nights per month) and sleep quality. Statistical analyses were performed using SPSS v26, including chi-square tests for categorical variables and multivariate logistic regression to adjust for potential confounders like age, BMI, smoking and physical activity. A p-value of <0.05 was considered statistically significant.

## Results:

This study revealed a significantly higher prevalence of both hypertension and arrhythmias among healthcare workers engaged in rotating or night shift schedules compared to those on fixed day shifts. Increased shift frequency, longer duration of night shift exposure, and poorer sleep quality were all significantly associated with elevated cardiovascular risk. Table 1 (see PDF) shows Demographic characteristics were largely comparable between the two groups. Night shift workers had a slightly higher mean age, BMI, and longer years of service. Table 2 (see PDF) shows Prevalence of hypertension was significantly higher in night shift workers compared to day shift counterparts. Table 3 (see PDF) shows Arrhythmia prevalence was significantly higher among night shift workers, particularly in those with >5 years of rotating shifts. Table 4 (see PDF) shows Night shift workers exhibited more frequent ventricular and supraventricular arrhythmias. Table 5 (see PDF) shows Longer duration of night shift exposure was significantly correlated with higher prevalence of hypertension and arrhythmia. Table 6 (see PDF) shows Poor sleep quality, as measured by PSQI, were significantly more prevalent in night shift workers and were associated with both hypertension and arrhythmia. Table 7 (see PDF) shows A significant positive correlation was found between frequency of night shifts per month and the occurrence of both hypertension and arrhythmia. Table 8 (see PDF) shows Multivariate logistic regression confirmed that night shift work, poor sleep and high BMI were independent predictors of both hypertension and arrhythmia. Table 9 (see PDF) shows Night shift work, longer shift duration (>5 years), and poor sleep quality were all significantly associated with higher odds of developing arrhythmia. Night shift workers had over 3 times the risk, while prolonged shift duration and poor sleep nearly doubled the risk. Table 10 (see PDF) shows Day shift workers had better average sleep duration and sleep efficiency than night shift workers.

## Discussion:

This analytical study demonstrates a significant association between night or rotating shift work and increased prevalence of hypertension and cardiac arrhythmias among healthcare workers. The findings align with previous literature emphasizing the detrimental cardiovascular effects of circadian rhythm disruption [7]. Night shift workers had notably higher blood pressure readings, greater arrhythmic events and more frequent poor sleep quality, all contributing to a composite cardiovascular risk profile [8]. The relationship was dose-dependent, with longer duration and higher frequency of night shifts correlating with more adverse outcomes. Physiologically, disruption of the endogenous circadian system can impair vascular tone regulation, autonomic balance, and hormonal rhythms, all of which play roles in the pathogenesis of hypertension and arrhythmogenesis [9]. Furthermore, the increased BMI and reduced physical activity levels among night shift workers compound the cardiovascular risks. Significantly, the study discovered that night shift workers had a much higher prevalence of sinus tachycardia and premature contractions, suggesting increased sympathetic activity and cardiac instability that is probably made worse by exhaustion and sleep debt [10]. Even after accounting for confounding factors including age, smoking status, and physical inactivity, the relationships that were found remained strong. This implies that shift work, especially night duty, is an occupational component that independently adds to cardiovascular strain. Furthermore, exposure to night shifts, poor sleep, and elevated body mass index were validated by logistic regression analysis as independent predictors of arrhythmia and hypertension [11]. These results highlight the necessity of policy-level measures to improve shift scheduling in medical facilities. Cardiovascular screening procedures, requiring sufficient recuperation times, and limiting the number of night shifts could significantly reduce long-term health hazards. Workplace wellness programs that encourage stress reduction, physical exercise, and good sleep hygiene may also be protective measures [12]. However, causal inference is limited due to the cross-sectional form of the study. It would be useful to conduct longitudinal research to monitor incidence over time and see whether shift alterations have reversible effects. However, the results show that this vital workforce is occupationally vulnerable, which supports the need for behavioral and structural precautions in healthcare settings.

## Conclusion:

The prevalence of hypertension and arrhythmias is much higher among healthcare workers who work night and rotating shifts. Longer exposure to night shifts and poor sleep quality were found to be powerful independent indicators. These data underline the critical need for workplace interventions to safeguard the cardiovascular health of healthcare workers who work shifts.
